# Therapeutic potential of TMSC-Exo for non-alcoholic fatty liver disease using the liver-on-a-chip model

**DOI:** 10.1515/jtim-2026-0007

**Published:** 2026-02-13

**Authors:** Shujiao He, Kexin Wang, Binghui Li, Wei Fang, Xinyi Wei, Fen Yao, Nan Wang, Xiaoxia Wang, Ying Zhang, Yi Gao, Yang Li, Shao Li, Shuqin Zhou, Juan Du, Qing Peng

**Affiliations:** General Surgery Centre, Department of Hepatobiliary Surgery II, Institute of Regenerative Medicine, Zhujiang Hospital, Southern Medical University, Guangzhou, Guangdong Province, China; Central Laboratory of The Second Affiliated Hospital, School of Medicine, The Chinese University of Hong Kong, Shenzhen & Longgang District People’s Hospital of Shenzhen, Shenzhen, Guangdong Province, China; School of Medicine, The Chinese University of Hong Kong, Shenzhen, Guangdong Province, China; Department of Pharmacology, Shantou University Medical College, Shantou, Guangdong Province, China; Department of Nutrition and Food Hygiene, Lanzhou University School of Public Health, Lanzhou, Gansu Province, China; Department of Anesthesiology of The Second Affiliated Hospital, School of Medicine, The Chinese University of Hong Kong, Shenzhen & Longgang District People’s Hospital of Shenzhen, Shenzhen, Guangdong Province, China

**Keywords:** non-alcoholic fatty liver disease, liver-on-a-chip, resmetirom, immortalized human mesenchymal stem cell-derived exosomes, proteomics

## Abstract

**Background and Objectives:**

Non-alcoholic fatty liver disease (NAFLD) has become a growing global public health concern. Effective therapeutic strategies for NAFLD remain urgently needed. Liver-on-a-chip (LC) technology offers an innovative platform for NAFLD modeling and drug development. This study aimed to develop a biomimetic liver-chip using co-cultured human hepatocyte (HepaRG) with hepatic stellate and endothelial cells to model NAFLD, and evaluate the therapeutic potential of scalable telomerase reverse transcriptase (hTERT)-immortalized umbilical cord mesenchymal stem cell-derived exosomes (TMSC-Exo).

**Methods:**

HepaRG cells, hepatic stellate cells, and endothelial cells were used to construct a dual-chamber biocompatible LC. The NAFLD model was induced by free fatty acid (FFA) and applied to evaluate the efficacy of resmetirom and TMSC-Exo for the treatment of NAFLD. Moreover, the high-fat (HF) diet-induced mouse model was analyzed to verify the in vitro results. Proteomic analyses were performed to explore the molecular mechanisms involved in the development of NAFLD and the effect of TMSC-Exo in treating NAFLD.

**Results:**

Cells cultured in LC showed better viability compared to those in the Transwell system. The on-chip NAFLD model mimicked the characteristics of NAFLD *in vivo*, including intracellular lipid accumulation and impaired hepatocyte functions in albumin synthesis, levels of urea, CYP1A2, and CYP3A4. Both TMSC-Exo and resmetirom displayed a significant effect in reducing the lipid accumulation in the on-chip NAFLD model. The TMSC-Exo showed superior effects in elevating the levels of albumin, urea, CYP1A2, and CYP3A4. The therapeutic effects of TMSC-Exo were also confirmed in the NAFLD mouse models. Proteomic analysis found that the top 15 up- and down-regulated differentially expressed proteins in NAFLD models compared to the control group were mainly associated with lipid metabolism, endoplasmic reticulum stress, and inflammation.

**Conclusions:**

Our on-chip NAFLD model successfully recapitulated key pathological features of hepatic steatosis and functional impairment. Using this model, we evaluated TMSC-Exo and demonstrated its significant therapeutic efficacy against NAFLD.

## Introduction

Non-alcoholic fatty liver disease (NAFLD) represents a prevalent public health concern, currently affecting approximately 30% of the global population.^[1,2]^ NAFLD comprises a spectrum of metabolic disorders in the liver, including hepatocytic lipid deposition, hepatic cell injury, and fibrosis. Despite its widespread prevalence, there exists a substantial gap in the comprehension of the pathophysiological mechanisms underlying NAFLD, alongside a paucity of effective pharmacological treatments. This is partly attributable to the lack of robust human NAFLD models that are essential for facilitating drug development.^[3]^ To date, only one medication (resmetirom) has been approved by the FDA on March 14, 2024 for the treatment of adults with noncirrhotic nonalcoholic steatohepatitis (NASH), a severe manifestation of NAFLD (https://www.fda.gov/news-events/press-announcements/fda-approves-first-treatment-patients-liver-scarring-due-fatty-liver-disease). Consequently, there remains an urgent need for further research into NAFLD and the development of effective therapeutic strategies.

Traditional preclinical studies have predominantly utilized two-dimensional (2D) cell cultures to explore the mechanisms underlying liver diseases and assess the efficacy of pharmacotherapeutics. However, 2D cell culture models are inadequate in replicating the three-dimensional (3D) complexity and cellular diversity inherent to hepatic tissues.^[4,5]^ In recent years, microfluidic organ-on-a-chip devices emerged as a new platform that offers an enhanced ability to replicate the microenvironment of actual organs/tissues, encompassing cell-to-cell interactions and extracellular matrix components. The microfluidic organ-on-a-chip devices can facilitate precise control over fluid flow and shear stress, thereby enabling an accurate simulation of physiological responses during drug testing. ^[4,5]^ Many efforts have been made to use microfluidic organ-on-a-chip to study NAFLD.^[6, 7, 8]^ In the previous studies, the liver tumor cell line HepG2 was frequently employed in the development of NAFLD models.^[7, 9, 10, 11, 12]^ However, the physiological functions, such as the metabolizing enzymes of HepG2, are significantly inferior to those of human primary hepatocytes.^[13,14]^ Furthermore, previous studies often used mono-cultured hepatocytes, which constrained the precise modeling of NAFLD’s pathological characteristics.^[9,15,16]^ This underscores the urgent need for a more biomimetic biochip that replicates the functionalities and interactions of liver cells, including hepatocytes, hepatic stellate cells (HSCs), and liver sinusoidal endothelial cells (LSECs), and accurately mimics the pathological features of NAFLD.

HepaRG is a human hepatic progenitor cell line with the ability to consistently differentiate into highly functional hepatocyte-like cells.^[17,18]^ It exhibits superior functionality compared to HepG2 cells and is increasingly utilized as an alternative to primary human hepatocytes for liver disease models.^[5,19,20]^ Previous studies have demonstrated that co-culture of cells enables better cell-cell interactions and physiological microenvironments compared to monoculture.^[21, 22, 23]^ To develop a biomimetic liver-on-a-chip (LC) model, this study utilized the HepaRG cell line in co-culture with HSCs and LSECs to recapitulate the liver’s minimal functional unit. NAFLD was simulated by introducing free fatty acids into the chips.

Recent studies have demonstrated that MSC-derived exosomes (MSC-Exo) can ameliorate NAFLD by modulating inflammation, regulating lipid metabolism, reducing endoplasmic reticulum stress and improving mitochondrial damage.^[24, 25, 26]^ However, their path to clinical application is fraught with challenges. A critical impediment to pharmaceutical manufacturing is the procurement of scalable MSC populations with stable, high-quality attributes. The expansion process is plagued by cellular senescence and diminishing potency, likely a consequence of telomere shortening. Compounding this issue is the heterogeneity among donor-derived MSCs, which introduces substantial batch-to-batch variation in the resulting cells and exosomes, thereby constraining clinical applicability.^[27]^ Our laboratory previously established a telomerase reverse transcriptase (hTERT)-immortalized umbilical cord-derived mesenchymal stem cell line (TMSC). This cell line can generate large quantities of exosomes with stable characteristics for at least 35 passages, representing a promising approach for future scalable exosome production.^[28]^ TMSC-derived exosomes (TMSC-Exo) have been shown to be effective in treating acute liver failure.^[28]^ However, the therapeutic potential of TMSC-Exo for NAFLD remains unknown. Using the on-chip NAFLD models, we compared the therapeutic efficacy of TMSC-Exo and resmetirom, the current medication available for NAFLD treatment. In addition, the efficacy of TMSC-Exo was confirmed using a NAFLD mouse model. Our findings indicate that TMSC-Exo exhibits promising effects in alleviating NAFLD compared to resmetirom.

## Materials and methods

### Cell sources and culture

HepaRG, EA. hy926 cells (a human umbilical vein fusion cell line) and LX2 cells (a human hepatic stellate cell line) used in this study were preserved by our laboratory. HepaRG cells were induced to differentiate before being introduced into the chip, as previously reported.^[8,29]^ HepaRG, EA. hy926 and LX2 cells were co-cultured in William’s E medium (Gibco, USA) supplemented with 10% fetal bovine serum (Gibco, USA), 1% penicillin-streptomycin antibiotics (Gibco, USA), 2 mmol/L glutamine (Thermo Fisher Scientific, MA, USA), 5 μg/mL insulin (MedChemExpress, USA), and 50 μmol/L hydrocortisone succinate (MedChemExpress, USA). TMSC (established by our team previously) was cultured in the AM-V Serum Free Medium (SC2013-G, Tianjin Haoyang Biotechnology Co., Ltd., China).

### Fabrication of liver-on-a-chip

**Figure 1:**
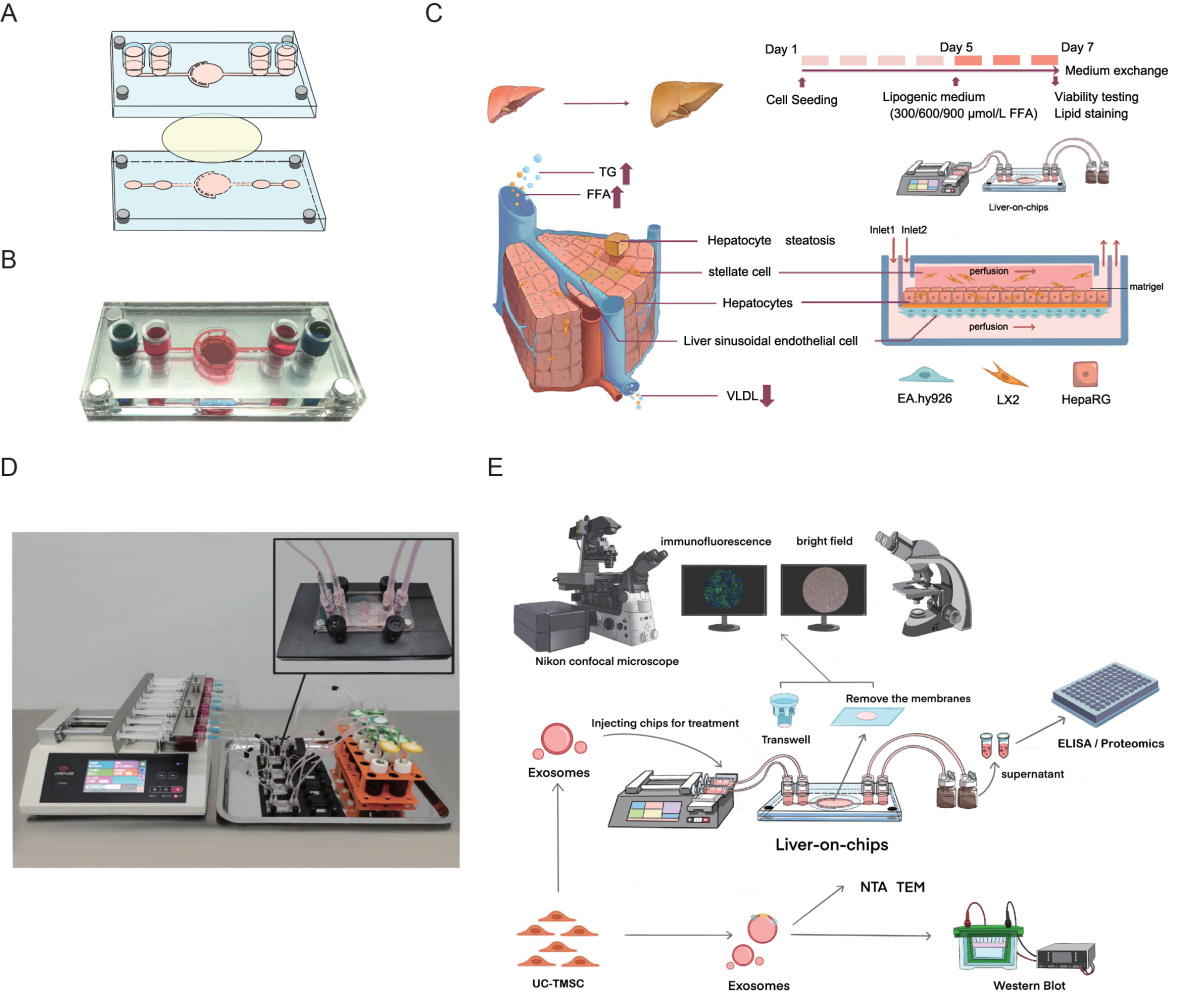
Procedure of the liver-on-a-chip for developing the NAFLD model and drug evaluation. (A) The schematic diagram of the chip. The chip consists of two culture chambers connected by polyester film to support the co-culture of hepatocytes and hepatic interstitial cells. (B) The real chip. (C) Schematic illustration of a liver function unit and the liver-on-a-chip for developing the NAFLD model. (D) Experimental setup of the dynamic perfusion system. (E) Liver-on-a-chip allows for cells in situ perfusion culture and functional analysis for drug evaluation. NAFLD: non-alcoholic fatty liver disease; TG: triglycerides; FFA: free fatty acid; VLDL: very low-density lipoprotein; TEM: transmission electron microscopy.

The microfluidic chip was designed and manufactured by Mesobiosystem Co., Ltd (Wuhan, China). As shown in Figures 1A and 1B, the microfluidic chip is composed of four distinct layers. The channels located on the top layer serve as the inlet and outlet for sample flow and are fabricated from polycarbonate (PC). The second and third layers comprise the flow channel, with dimensions of 0.5 mm in width and 0.5 mm in depth, and the detection chamber, measuring 8 mm in diameter and 1 mm in depth. These layers are constructed using photolithography and polydimethylsiloxane (PDMS) curing molding techniques. The fourth layer functions as the substrate layer, with dimensions of 55 mm in length and 25 mm in width, and is also manufactured from PC. Before experimentation, the microfluidic chips and silicone tubing underwent autoclave sterilization to ensure sterility. To mimic the hepatic blood sinusoids within the chip, HepaRG, LX2, and EA. hy926 cells were cultured on both the upper and lower surfaces of a polyester membrane, which was placed between the two chambers of the second and third layers (Figure 1C). EA.hy926 cells, at a concentration of 1 × 10^5^ cells/mL, were embedded in a 20% Matrigel matrix and seeded onto the membrane surface of the lower PDMS layer. HepaRG cells (2 × 10^5^ cells/ml) and LX2 cells (2 × 10^4^ cells/mL) were embedded in 20% Matrigel gel and seeded on the surface of the membrane of the top PDMS layer. The cells within the chip were cultured in medium under conditions of 37 °C and 5% CO_2_. Dynamic perfusion was performed using a micro syringe pump (perfusion rate at 60–100 μl/h). The cellular metabolites were collected for downstream analysis, such as the ELISA assay (Figure 1D and 1E). The corresponding Transwell group was designated as a control group for the LC. The culture medium in the Transwell device was refreshed every 24 h.

### Cell viability assay

During the co-culture period, cell morphology was observed by an inverted phase contrast microscope (Olympus, Tokyo, Japan), and cell viability was assessed by the LIVE/ DEAD Viability/Cytotoxicity Kit (Keygen Biotech Co., Ltd., Jiangsu, China), which employs Calcein-AM at a concentration of 2 μmol/L for staining live cells and propidium iodide (PI) at a concentration of 8 μmol/L for staining dead cells. Cell nuclei were stained with 4’,6-diamidino-2-phenylindole (DAPI) stain (Solarbio, C0065). Observations were conducted using a confocal microscope (Nikon, Tokyo, Japan). Cell viability was determined based on the staining results from Calcein-AM and DAPI.

### Immunofluorescence staining

To fix the cells, 4% paraformaldehyde was used for 15 min at room temperature. After permeabilizing the cells with 0.1% Triton X-100, the cells were incubated with 0.5% Triton X-100 and 5% BSA in PBS for 1 h. The cells were then incubated with the primary antibodies albumin (ALB, Abcam, ab207327, Rabbit, 1:500), alpha-fetoprotein (AFP, Abcam, ab133617, Rabbit, 1:50), CD31 (Cell signaling technology, 3528, Mouse, 1: 800), zonula occludens-1 (ZO-1, Abcam, ab221547, Rabbit, 1:500) and VE-Cadherin (Proteintech, CL594-66804, Mouse, 1: 50) overnight at 4 °C. Finally, the cells were incubated with the corresponding secondary antibodies (Goat Anti-Mouse IgG conjugated with Alexa Fluor 488(Proteintech, SA00013-1), Goat Anti-Rabbit IgG conjugated with Alexa Fluor 594(Proteintech, SA00013-4) at room temperature for 1 h. DAPI was used for staining the nuclei. A confocal microscope (Nikon) was used for imaging.

### ELISA assay

Albumin, urea, total bile acids, CYP1A2, and CYP3A4 levels were measured using an enzyme-linked immunoassay kit (Shanghai Enzyme-linked Biotechnology, China), following the manufacturer’s protocols. The absorbance of the samples was measured using a microplate reader (Multiskan FC, Thermo Fisher Co., Ltd., USA).

### Induction of NAFLD

A medium containing free fatty acids (FFA) was formulated by combining palmitic acid and oleic acid, both sourced from Sigma-Aldrich (MO, USA), in a 1:2 ratio, achieving final concentrations of 300 μmol/L, 600 μmol/L, and 900 μmol/L, as previously documented.^[8]^ The cells cultured in LC or Transwell systems were exposed to the medium containing FFA for a duration of 72 h to induce NAFLD. In contrast, the control group was maintained in a standard medium for the entire 7-day period.

### Lipid droplet staining and quantification

For visualization of intracellular lipid accumulation induced by FFA, cells were stained with Nile Red (Solarbio, China). Briefly, the cells were fixed in a 4% formaldehyde solution for 1 h at room temperature. Subsequently, lipid droplets and nuclei were stained using 2 μmol/L Nile Red and 10 μg/mL DAPI, respectively, both diluted in a solution containing 0.5% Triton X-100 and 5% BSA in PBS for 1 h. Imaging was performed using a confocal microscope (AX NIS-Elements 5.4, Nikon, Japan), and the resulting images were analyzed with ImageJ software. The Nile Red fluorescence intensity was measured, normalized by cell area, and expressed as a fold change relative to the control group.

### Isolation and identification of TMSC-derived exosomes

TMSCs were constructed and characterized by our team as previously described.^[28]^ TMSC-Exo was isolated from passages 5 to 15 of TMSCs using molecular size exclusion chromatography (SEC). Culture supernatants were harvested when TMSCs reached approximately 90% confluence and subsequently centrifuged at 2000×*g* for 20 min at 4 °C. The resulting supernatants were filtered through a sterile 0.22 μm membrane (Millipore, Billerica, MA, USA). The filtrates were then subjected to ultrafiltration using a 100-kDa hollow fiber membrane tube (Millipore, USA) at 4 °C and 3215 ×*g* for 30 min to concentrate the supernatant enriched with TMSC-Exo. The TMSC-Exo was further purified using the Exosupur^®^ small extracellular vesicle purification kit (Echobiotech Co., Ltd., Beijing, China) and stored at −80 °C. A BCA Protein Assay Kit (Beyotime, Shanghai, China) was used to measure protein concentrations.

TMSC-Exo was analyzed by single particle interferometric reflectance imaging sensing (SP-IRIS) using the ExoView platform (Nanoview, USA).^[28]^ Vesicle diameter and number were evaluated by a Flow NanoAnalyzer N30 (NanoFCM, Xiamen, China). Expression of exosome markers CD63, CD81, and CD9, and ER protein Calnexin was analyzed by Western blotting. TMSC-Exo was visualized using transmission electron microscopy (TEM; Hitachi, Japan).

### Drug testing

Two drugs were selected to assess the therapeutic effect of NAFLD: Resmetirom (HY-12216; MedChemExpress, USA) and TMSC-Exo. Based on previous studies,^[30]^ the optimal working concentrations for resmetirom were determined to be 100 μmol/L, TMSC-Exo was used at 400 μg/mL and 800 μg/mL. TMSC-Exo distribution was investigated by monitoring the PHK26 fluorescent dye using by confocal microscope for hepatic cells in the LC. After 72 h, at the end of the treatment, the culture compartment of the LC was fixed with 4% PFA, and nuclei were stained with DAPI for imaging processes.

To test the therapeutic effect of drugs, cells in the LC group were exposed to a culture medium containing 600 μmol/L FFA for 72 h to induce NAFLD. Subsequently, these cells were treated with culture medium, either supplemented with the drug or not, for an additional 48 h. In contrast, the control group was maintained in normal culture medium throughout the entire 7-day period. On the 7th day, assessments were conducted to evaluate cell viability, lipid accumulation, and liver cell function.

### Animal experiments

Five-week-old male C57BL/6J mice (body weight 20 ±1 g; Jiangsu Gempharmatech Co., Ltd.) were housed in cages (three animals per cage) under controlled environmental conditions (relative humidity 60%–70%; temperature 23 °C ± 1 °C). All the procedures of animal experimentation were approved by the Animal Ethics Committee of Shenzhen Longgang District People’s Hospital (approval number: 2024027DW). In animal studies, ARRIVE guidelines are followed.^[31]^

Mice were randomly divided into four groups (*n* = 5 for each group). A normal diet (10% trans-fat; PiclLab5053, LabDiet, USA) was fed to one group of mice, and three other groups were given high-fat (HF) (60% trans-fat) (D12492, Research Diets, USA) diets to induce obesity (DIO). After HFD treatment (12 weeks), one group of DIO mouse was given resmetirom (3 mg/ kg) thrice weekly for 4 weeks (NAFLD + resmetirom group),^[32]^ one group of DIO mouse was given TMSC-Exo (10 mg/ kg) thrice weekly (NAFLD + TMSC-Exo group) for 4 weeks, whereas the normal diet-treated mouse and other group of DIO mouse were given PBS in equal volumes (Normal or NAFLD group) for 4 weeks.

At week 16, an intraperitoneal glucose tolerance test (IPGTT) was conducted as previously described.^[32]^ It was decided to sacrifice mice at the end of the trial. The serum levels of alanine transaminase (ALT), aspartate aminotransferase (AST), total cholesterol (TC), and triglycerides (TG) were determined using corresponding assay kits (Shanghai Enzyme-linked Biotechnology, China). Fresh liver tissues were collected and stored at –80 °C.

### In vivo bioluminescence imaging

A technique for assessing biodistribution of exosomes *in vivo* was applied, which involved staining them with Exosome Fluorescent labeling dye (DiR) according to the manufacturer’s instructions to obtain suspensions of TMSC-Exo labeled with DiR (TMSC-ExoDiR). TMSC-ExoDiR were treated at 50 μg/100 μL per mouse. After 24 h of treatment, the fluorescence signals from TMSC-ExoDiR in mice were evaluated by bioluminescence imaging. The mice were euthanized immediately after the trial, and the heart, lungs, liver, kidneys, and skin were imaged.

### Histological staining

To detect pathological changes and lipid accumulations in the liver. Liver samples were immersed in 4% paraformaldehyde (Solarbio), embedded in paraffin, and sectioned into 4 μm slices. The sections were stained with hematoxylin and Eosin (H&E) and Oil Red O (Solarbio), following standard protocols.

### Proteomics analysis

To investigate the mechanisms underlying the progression of NAFLD and the inhibitory effects of TMSC-Exo on NAFLD. Samples from the control group (without FFA induction), the on-chip NAFLD group, and the TMSC-Exo treatment group (*n* = 4 for each group) were subjected to label-free quantitative proteomics as described previously.^[33]^ Briefly, protein extraction and digestion for the HepaRG and LX2 cell samples were conducted using the filter-aided sample preparation (FASP) method.^[34]^ Protein concentration was measured by spectrophotometry using a Nanodrop 2000c Spectrophotometer (USA). The digestive peptides of each sample were desalted on C18 Cartridges (Reprosil, Germany). Next, LC-MS/MS analysis was performed on a Thermo Scientific Orbitrap Exploris 480 MS with Easy-nLC 1200. The peptides were loaded onto a C18 column (100 μm I.D., 2 cm length, 3μm particle size) with a flow rate of 300 nL/ min using solution A (0.1% FA in water) and solution B (0.1% FA in 80% ACN). The separation gradient was 3%–8% B for 5 min, 8%–28% B for 58 min, 28%–40% B for 12 min, 40%–100% B for 5 min, 100% B for 5 min, and 100%–3% B for 3 min. The data were searched using a target-decoy approach against Uniprot Human (released 2023.10.16, 20,426 proteins) reference proteome (FASTA file) (FDR ≤ 0.01) at the level of proteins and peptides. The remaining proteins (after contaminating proteins were removed) were used for subsequent quantitative analyses. Analyses were performed with the GraphPad Prism Software Version 9.3.0. Protein quantitative data values were imported into Perseus for data standardization and filling of missing values with the principle of normal distribution (width = 0.3, downshift = 1.8), Principal Component Analysis (PCA), and Volcano Plot Analysis of Variance. A t-test was then used to calculate the extent to which enrichment was significant. *P* < 0.05 was considered significant for all the analyses.

### Western blot

Total protein of cells in each group was isolated using RIPA (Beyotime, China), and concentrations were determined using BCA (Beyotime). The protein samples were separated by SDS-PAGE (Beyotime) and transferred to NC membranes (Cytiva, Germany) for immunoblotting. The membrane was incubated with primary antibodies overnight at 4 °C, and then incubated with 2nd antibody (1:7000) for 1h at room temperature (RT). The intensity of protein bands was quantified with ImageJ software.

### Statistical analysis

Data were presented as mean ± standard deviation (SD) for at least three independent experiments. The significance level between the two groups was determined by a two-tailed unpaired Student’s *t*-test. Multiple group comparison was analyzed using a one-way analysis of variance (ANOVA) test. Statistical significance was indicated as ^*^ for *P* < 0.05, ^**^ for *P* < 0.01, ^***^ for *P* < 0.001, ^****^ for *P* < 0.0001 and ns for no significance.

## Results

### Construction and validation of the liver-on-a-chip

To model the physiological architecture and function of the liver, a microfluidic chip, namely LC, was designed. In this system, EA. hy926 cells were seeded on the lower surface of a polyester membrane within the chip to simulate the sinusoidal endothelial layer. Concurrently, HepaRG and LX2 cells were cultured on the upper surface of the membrane to emulate the liver plate. (Figure 1).

**Figure 2:**
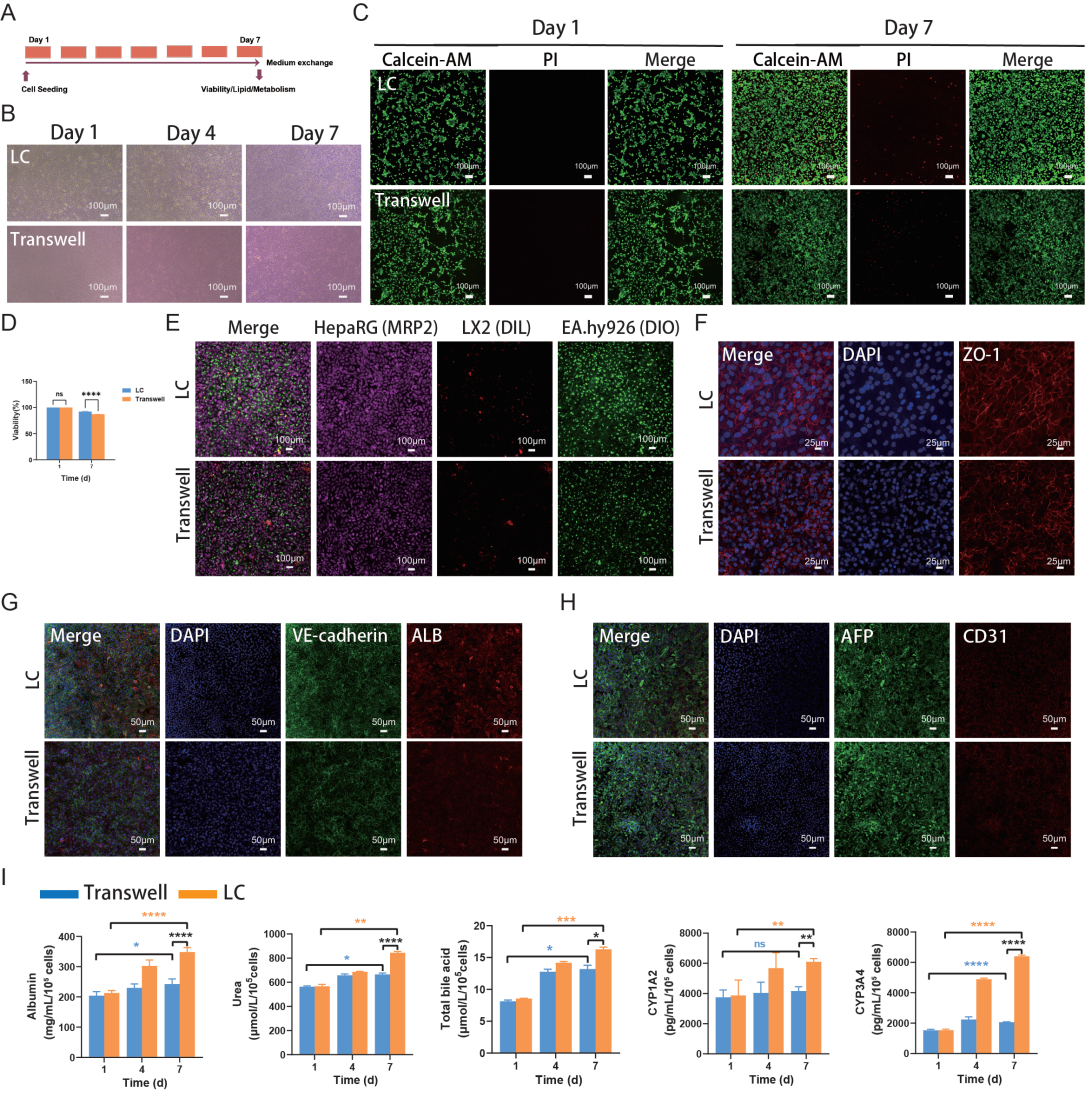
Analysis of cells in the liver-on-a-chip model. (A) Experimental timeline. (B) The morphology of cells on days 1, 4, and 7 (scale bar = 100 μm). (C) The viability of cells on days 1 and 7 (scale bar = 100 μm). (D) Quantification of cell viability. (E) The identified the co-culture cells, hepaRG cells (far-red), LX2 (red), and EA.hy926 cells (green) (scale bar = 100 μm). (F) The expression of ZO-1 (red) on endothelial cells on day 7 (scale bar = 25 μm). (G) The expression of ALB (red) and VE-Cadherin (green) on day 7 (scale bar = 50 μm). (H) The expression of AFP (green) and CD31 (red) on day 7 (scale bar = 50 μm). (I) The levels of albumin, urea, total bile acid, CYP1A2, and CYP3A4 were measured by ELISA. ^*^P < 0.05, ^**^P < 0.01, ^***^P < 0.001, ^****^P < 0.0001, and ns indicates not significant. LC: liver-on-a-chip; ELISA: enzyme-linked immunosorbent assay; DAPI: 4’,6-diamidino-2-phenylindole; ALB: albumin; AFP: alpha-fetoprotein.

The morphology, viability, and function of cells were observed after 7 d of coculture (Figure 2A). The cells maintained normal morphology on day 7 (Figure 2B). In the LC group, cell viability remained high throughout the 7-day culture period (Figure 2C). There was no difference in cell viability between the on-chip and Transwell cultures on day 1. By day 7, however, viability in the LC (92%) was slightly higher than that in the Transwell system (87%) (Figure 2C and 2D). Figure 2E demonstrated the successful coculture of HepaRG, LX2, and EA. hy926 cells within the LC. Fluorescence analysis revealed a significantly higher expression of ZO-1 in EA. hy926 cells within the LC compared to the Transwell group (Figure 2F). The functional hepatocyte marker, albumin (ALB), produced by HepaRG cells, was notably elevated in the LC, whereas the level of alpha-fetoprotein (AFP) remained comparable to that in the Transwell model (Figure 2G and 2H). ELISA data indicated that the concentrations of ALB and CYP3A4 in the LC group on days 4 and 7 were significantly higher than those in the Transwell group **(**Figure 2I**)**. Furthermore, LC culturing led to a significant increase in the levels of urea, total bile acid (TBA), and CYP1A2 in hepatocytes on day 7 compared to the Transwell group **(**Figure 2I**)**. These findings suggest that hepatocytes cultured in the LC exhibit enhanced functional capacity relative to those in the Transwell system.

### Establishing the NAFLD model on the chip

**Figure 3:**
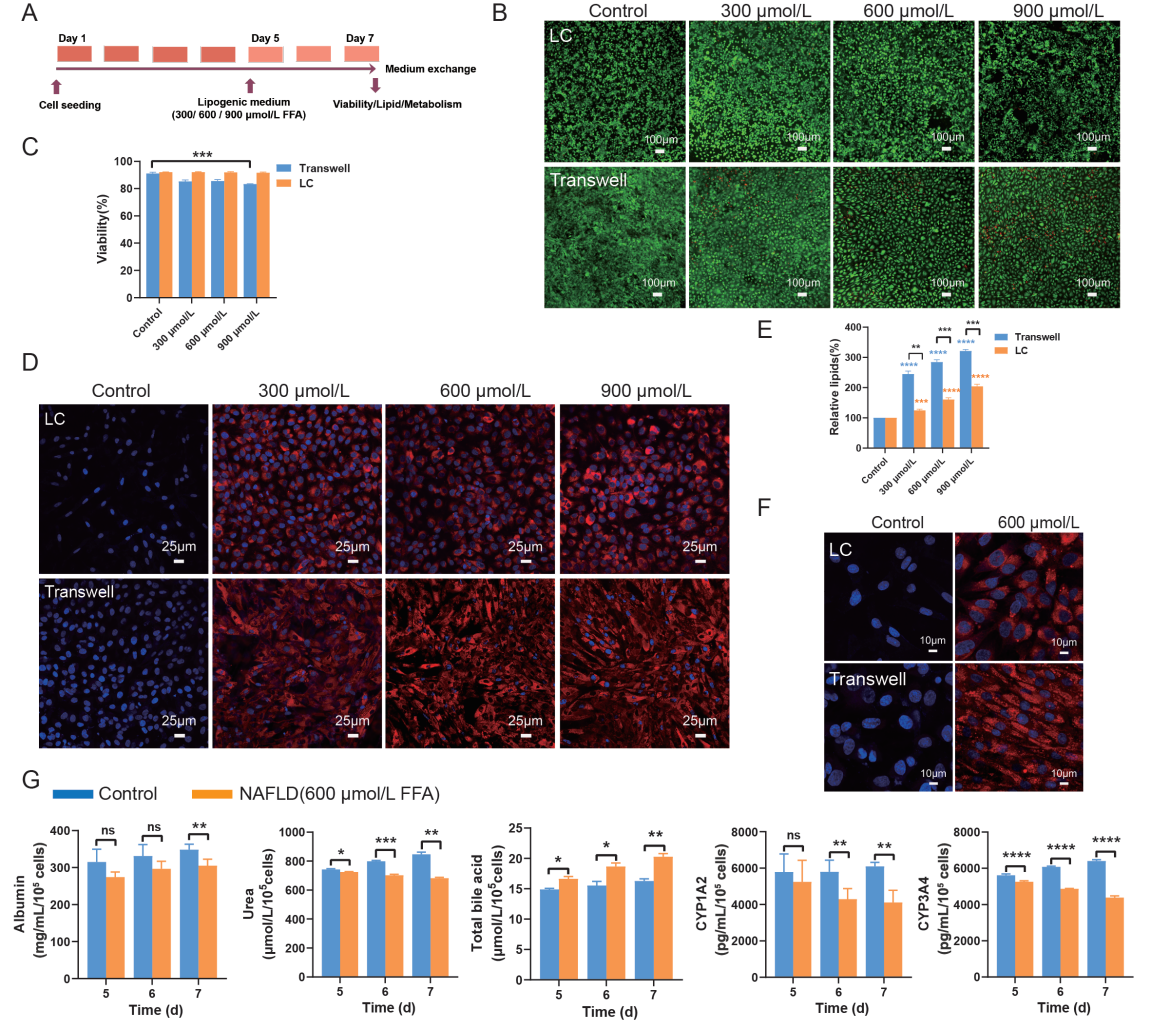
Characterization of the liver-on-a-chip NAFLD model. (A) Experimental timeline of FFA-induced NAFLD. (B) The viability of cells after FFA inducing (scale bar = 100 μm). (C) Quantification of cell viability. (D) Visualized lipid accumulation with different FFA concentrations using Nile Red (red) staining (scale bar = 25 μm). (E) Quantification of lipid accumulation by the volume ratio of lipids to nuclei. (F) Magnified view of cellular lipid droplets (scale bar = 10 μm). (G) The levels of albumin, urea, total bile acid, CYP1A2, and CYP3A4 were measured by ELISA. ^*^P < 0.05, ^**^P < 0.01, ^***^P < 0.001, ^****^P < 0.0001, and ns indicates not significant. NAFLD: non-alcoholic fatty liver disease; FFA: free fatty acid. LC: liver-on-a-chip; ELISA: enzyme-linked immunosorbent assay.

Cell viability, lipid accumulation, and cell function were measured in hepatic cells treated with FFA ranging from 300–900 μmol/L (Figure 3A). At 900 μmol/L FFA treatment, cell viability was significantly reduced in the Transwell system relative to the LC group. However, no statistically significant differences were detected between the LC and Transwell groups under other concentration conditions. (Figure 3B and 3C). Lipid staining with Nile Red revealed a dose-dependent increase in lipid accumulation with higher FFA concentrations **(**Figure 3D and 3E**)**. Notably, intracellular lipid accumulation at each concentration of FFA within the LC was significantly lower than that in the Transwell device **(**Figure 3D and 3E**)**. Figure 3F showed that cell nuclei were surrounded by lipid droplets, resembling microvesicular steatosis seen *in vivo*.^[35]^ Treatment with 600 μmol/L FFA significantly reduced albumin (ALB) and urea levels, as well as the levels of CYP1A2 and CYP3A4, compared to the untreated control group (Figure 3G). In contrast, FFA elevated TBA levels compared to the control group (Figure 3G). These results demonstrated successful establishment of NAFLD models in both LC and Transwell systems, as evidenced by recapitulation of key pathological features, including steatosis and hepatocyte dysfunction. However, the LC model showed significantly less lipid accumulation than the Transwell system.

### Identification of TMSC-Exo

**Figure 4:**
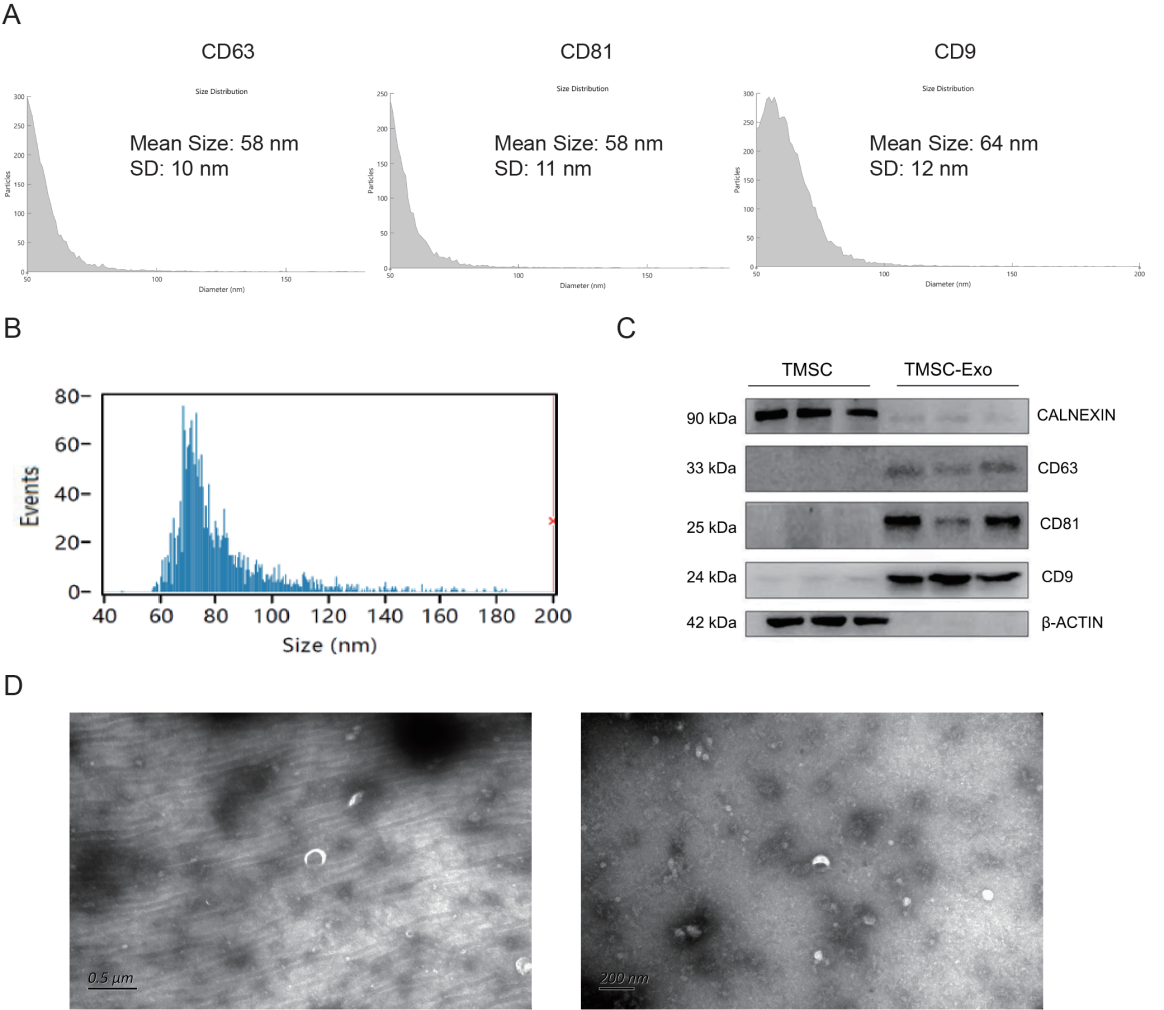
Characterization of TMSC-Exo. (A) NanoView analyzer for CD63, CD81, and CD9 expression in TMSC-Exo. (B) FCM analyzer for the particle size of TMSC-Exo. (C) Western blot for exosome marker protein CD63, CD81, and CD9 expression in TMSC-Exo. (D) TEM to evaluate the morphology of TMSC-Exo (left: scale bar = 0.5 μm; right: scale bar = 100 nm). FCM: flow cytometry; TEM: transmission electron microscope.

TMSC-Exo were identified for size, surface markers, and morphology using NanoFCM, NanoView Analyzer, Western blotting, and TEM, respectively. NanoView analysis confirmed the presence of exosomal markers CD63, CD81, and CD9 (Figure 4A). NanoFCM results showed that the average diameter of TMSC-Exo was 74.75 nm (Figure 4B). Western blotting analysis validated the expression of CD63, CD81, and CD9, while confirming the absence of Calnexin (Figure 4C). TEM imaging revealed a characteristic spherical or saucer-like morphology with a double-layered membrane structure (Figure 4D). These results confirmed that TMSC-Exo isolated in this study exhibits typical exosomal features and expressed exosome-specific markers, meeting the established criteria for exosomes.^[36,37]^

### TMSC-Exo exhibited therapeutic potential for NAFLD in the LC model

**Figure 5:**
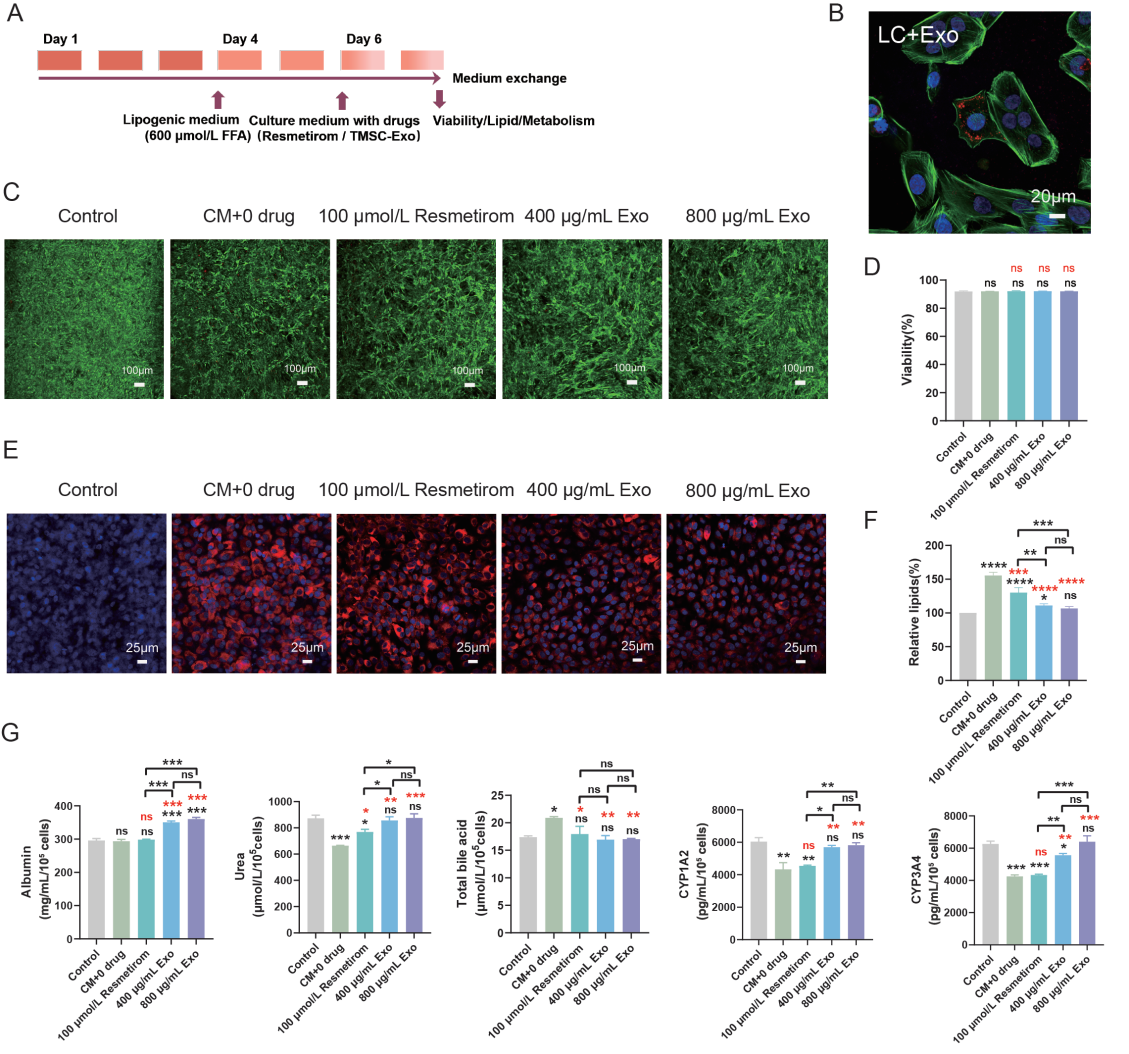
The liver-on-a-chip NAFLD responds to resmetirom and TMSC-Exo. (A) The protocol for drug evaluation. (B) TMSC-Exo distribution in hepatic cells was investigated by monitoring the PHK26 fluorescent dye (red) using by confocal microscope (scale bar = 20 μm). (C) Cell viability after adding resmetirom and TMSC-Exo (scale bar = 100 μm). (D) Quantification of cell viability after adding resmetirom and TMSC-Exo. (E) Lipid accumulation after treatment with resmetirom and TMSC-Exo (scale bar = 25 μm). (F) Quantification of lipid accumulation under different treatments. (G) The metabolites after adding resmetirom and TMSC-Exo. NAFLD: non-alcoholic fatty liver disease; CM: common medium; TMSC-Exo (Exo): immortalized mesenchymal stem cell-derived exosome (TMSC-derived exosome). ^*^P < 0.05, ^**^P < 0.01, ^***^P < 0.001, ^****^P < 0.0001, and ns indicates not significant. (*(black) *vs*. control group; *(red) *vs*. CM + 0 drug group).

To evaluate the utility of the LC NAFLD model for drug screening, we tested the therapeutic potential of resmetirom and TMSC-Exo (Figure 5A). Figure 5B showed the uptake of TMSC-Exo (red) by the on-chip cells (Figure 5B). Cell viability remained unaffected across all treatment groups, indicating that the tested drugs were non-toxic (Figure 5C and 5D). Nile Red staining demonstrated that both resmetirom and TMSC-Exo treatments (at either 400 or 800 μg/mL) effectively reduced lipid accumulation (Figure 5E and 5F). Both 400 μg/mL and 800 μg/mL concentrations of TMSC-Exo significantly elevated ALB levels, whereas resmetirom exhibited no significant effect on ALB levels in the NAFLD models. Furthermore, both treatments enhanced urea levels, with more pronounced effects observed in the TMSC-Exo groups. Compared to the control NAFLD group, both TMSC-Exo and resmetirom treatments reduced TBA synthesis. CYP1A2 and CYP3A4 levels were markedly increased following administration of TMSC-Exo (at concentrations of 400 μg/mL and 800 μg/mL), whereas resmetirom exhibited no significant effect in the NAFLD models (Figure 5G). Notably, no significant differences were observed in the change of these functional indicators at both MSC-Exo doses (400 μg/mL *versus* 800 μg/mL).

### TMSC-Exo significantly improved NAFLD in vivo

**Figure 6:**
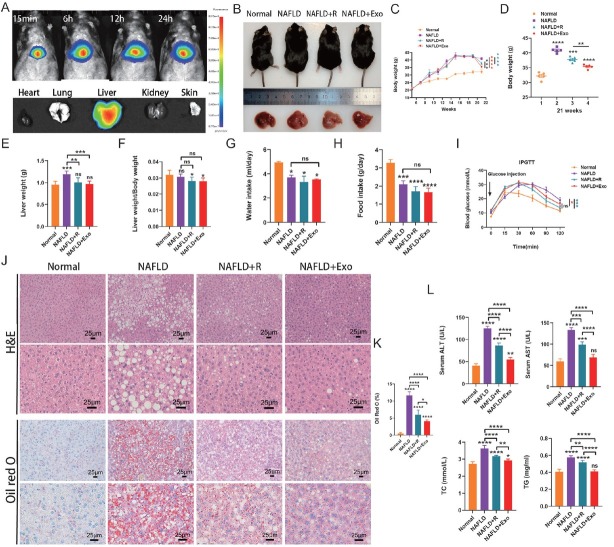
Characterization of NAFLD after resmetirom (R) and TMSC-Exo injections *in vivo*. (A) Exosomes homing *in vivo*. (B) Representative macroscopic mouse images and liver images. (C) Body weight curves. (D) Body weight at 21 weeks. (E) Liver weight. (F) The ratio of liver weight and body weight. (G) Water intake. (H) Food intake. (I) Blood glucose curves. (J) H&E and Oil Red O staining of liver tissues from control (*n* = 5), NAFLD (*n* = 5), resmetirom (*n* = 5), and TMSC-Exo (*n* = 5) groups mice (scale bar = 25 μm). (K) The positive area percentage of Oil Red O staining (*n* = 5) was calculated. (L) The levels of ALT, AST, TC, and TG were measured by ELISA. H&E: Hematoxylin and Eosin. ALT: alanine transaminase; AST: aspartate aminotransferase; NAFLD: non-alcoholic fatty liver disease; TG: triglyceride; TC: total cholesterol. ^*^P < 0.05, ^**^P < 0.01, ^***^P < 0.001, ^****^P < 0.0001, and ns indicates not significant.

To investigate the distribution of TMSC-Exo *in vivo*, the exosomes were labeled with PKH67 and administered *via* tail vein injection into mice. *In vivo* bioluminescence imaging (BLI) of liver tissue revealed that exosomes began to accumulate in the liver as early as 15 min post-injection, with signal intensity remaining elevated for up to 24 h. The imaging results indicated that exosomes predominantly accumulated in the liver, with some presence in the lungs, but were absent in the heart and kidneys (Figure 6A).

The therapeutic efficacy of TMSC-Exo and resmetirom in NAFLD was evaluated using a high-fat diet (HFD)-induced obese (DIO) mouse model, which exhibits severe hepatic steatosis and liver damage. NAFLD mice were randomly assigned to three groups: the DIO control group, the resmetirom-treated group, and the TMSC-Exotreated group, with treatments administered over 4 weeks. Representative gross images of the mice and their livers are presented in Figure 6B. Both resmetirom and TMSC-Exo treatments significantly reduced liver and body weights at the age of 21 weeks (Figure 6C-6F). There were no significant differences in daily food and water intake among the three groups (Figure 6G and 6H). The intraperitoneal glucose tolerance test (IPGTT) results demonstrated that both TMSC-Exo and resmetirom administration significantly improved HFD-induced glucose intolerance compared to the untreated DIO control group. No significant difference was observed between the TMSC-Exo-treated and resmetirom-treated groups (Figure 6I). The results of H&E and Oil Red O staining demonstrated that the DIO group exhibited a marked increase in lipid droplets. In contrast, both TMSC-Exo and resmetirom treatments decreased the lipid accumulation and alleviated the hepatic steatosis (Figure 6J and 6K). These effects were more pronounced in the TMSC-Exo-treated group (Figure 6J and 6K). Consistent with these findings, ELISA analyses demonstrated that both resmetirom and TMSC-Exo significantly attenuated the elevation of serum levels of ALT, AST, and TG, which are critical indicators of hepatic injury. Notably, TMSC-Exo treatment exhibited a more pronounced effect in improving ALT, AST, and TG levels. However, neither intervention resulted in an improvement in TC levels (Figure 6L). These results indicate that both resmetirom and TMSC-Exo contribute to the amelioration of NAFLD with favorable metabolic outcomes, with TMSC-Exo showing superior efficacy in reducing hepatic lipid accumulation and mitigating liver damage.

### Proteomic analysis

**Figure 7:**
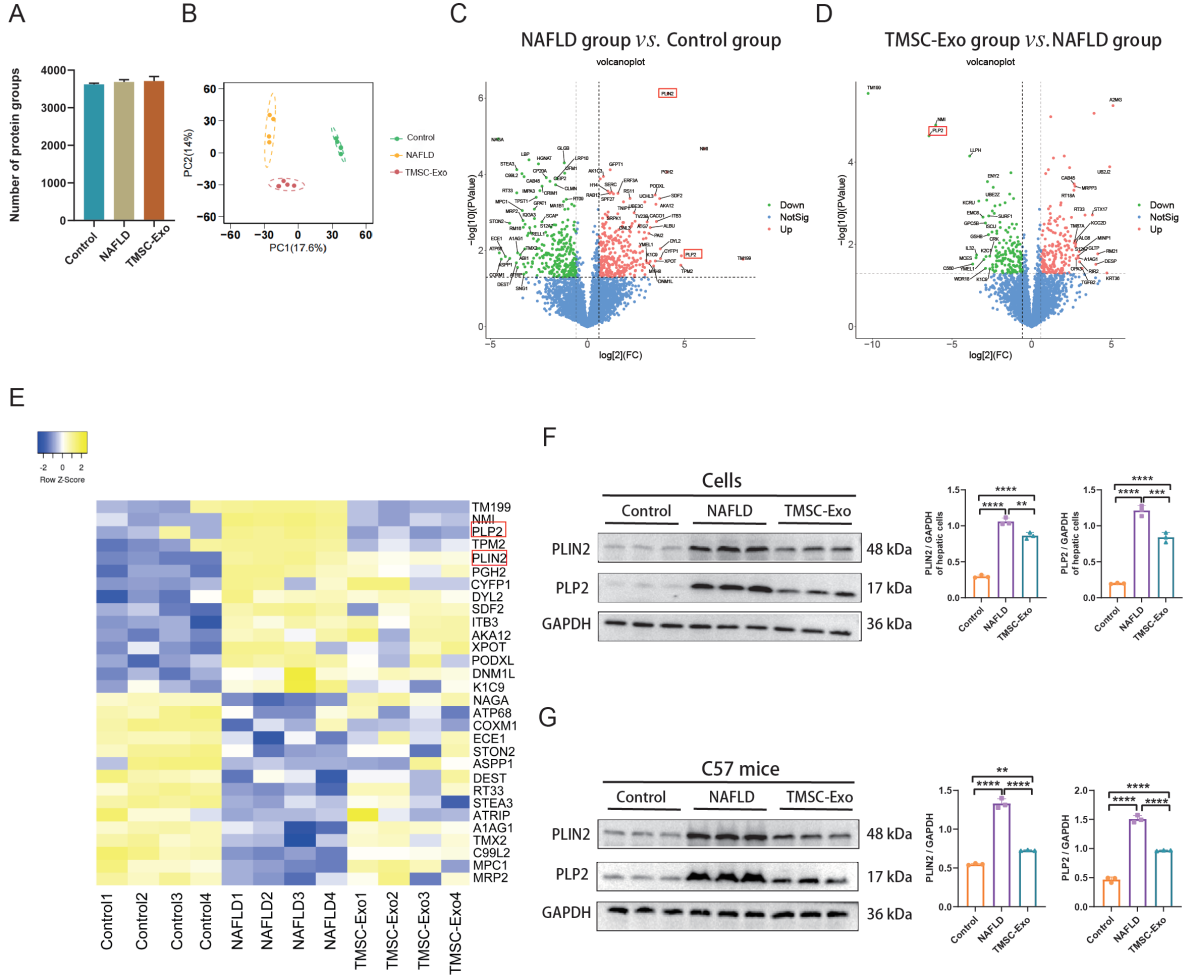
Label-free quantitative proteomics. (A) Comparison of the number of proteins identified in the control, NAFLD, and TMSC-Exo groups. (B) Principal component cluster analysis of protein samples. (C) Volcano plot for the top 20 DEPs in the NAFLD group (*n* = 4) and the control group (*n* = 4). (D) Volcano plot for the top 20 DEPs in the TMSC-Exo treatment group (*n* = 4) and NAFLD group (*n* = 4). (E) The heat map reflects the differential expression changes of different proteins in the control group (*n* = 4), NAFLD group (*n* = 4), and TMSC-Exo group (*n* = 4). (F) The proteins of PLIN2 and PLP2 in different groups were *in vitro* detected by Western blot, data are representative of three independent experiments (mean ± SD). (G) The proteins of PLIN2 and PLP2 in different groups were detected *in vivo* by Western blot, data are representative of three independent experiments (mean ± SD). ^*^P < 0.05, ^**^P < 0.01, ^***^P < 0.001, ^****^P < 0.0001, and ns indicates not significant. DEPs: differentially expressed proteins; NAFLD: non-alcoholic fatty liver disease.

Proteomic analysis identified 3,624, 3,688, and 3,710 proteins in the control, NAFLD, and TMSC-Exo treatment groups, respectively (Figure 7A). Principal component analysis (PCA) demonstrated a clear separation among the three groups (Figure 7B). In the NAFLD group, 306 proteins were found to be upregulated, while 189 proteins were downregulated in comparison to the control group (Figure 7C). Furthermore, following TMSC-Exo treatment, 138 proteins were upregulated and 155 proteins were downregulated relative to the NAFLD group (Figure 7D). The heat map presented in Figure 7E displayed the top 15 up- and down-regulated differentially expressed proteins (DEPs) in the NAFLD group compared to the control, along with their expression levels in the TMSC-Exo treatment group. These proteins include TM199, NMI, PLP2, TPM2, PLIN2, PGH2, CYFP1, DYL2, SDF2, ITB3, AKA12, XPOT, PODXL, DNM1L, K1C9, that were upregulated in the NAFLD group, and NAGA, ATP68, COXM1, ECE1, STON2, ASPP1, DEST, RT33, STEA3, ATRIP, A1AG1, TMX2, C99L2, MPC1, MRP2, that were down-regulated in the NAFLD group. Notably, both PLP2 and PLIN2 were reported to be up-regulated in NASH or NAFLD models in previous studies.^[38, 39, 40]^ They showed significant upregulation in the NAFLD group compared to controls, which was subsequently reversed by TMSC-Exo treatment (Figure 7E), suggesting their potential role in NAFLD pathophysiology. Western blot analysis confirmed these findings, demonstrating elevated PLP2 and PLIN2 expression in NAFLD and their downregulation after TMSC-Exo intervention in both *in vitro* and *in vivo* models (Figure 7F and 7G).

## Discussion

The development of a biomimetic LC model for NAFLD represents a significant advancement in the field of organ-on-a-chip technology.^[7,41]^ Using a four-layer microfluidic chip, our study successfully constructed an LC model with HepaRG, LX2, and EA. hy926 cells 3D co-culture that recapitulates key features of NAFLD, including hepatic steatosis and functional impairment.

NAFLD is characterized by alterations in lipid metabolism. Traditional 2D cell cultures and animal models have been widely used in NAFLD research, but they often fail to fully replicate the complex pathophysiology of human NAFLD. 2D cultures lack the three-dimensional architecture and cellular interactions present *in vivo*, while animal models are limited by interspecies differences. Our LC model addresses these limitations by incorporating multiple cell types (hepatocytes, hepatic stellate cells, and endothelial cells) in a dynamic microfluidic environment, which closely mimics the liver’s physiological microenvironment. The results demonstrated that those cells achieved higher viability and function on the LC than on the static Transwell model. This result may be attributed to the dynamic perfusion, which enhances the intercellular nutrient and signaling exchange. This finding is consistent with the results reported in other studies.^[42]^ Additionally, we constructed an NAFLD model on the LC using FFA. The data demonstrated that NAFLD-associated characteristics, such as lipid accumulation and impaired hepatocyte function, were successfully recapitulated within the LC NAFLD model. We observed improved cell viability, reduced lipid droplet accumulation, and delayed disease progression in the LC compared to the static Transwell model, aligning with previous findings.^[8]^ These superior outcomes likely stem from the LC’s optimized nutrient perfusion dynamics, which not only sustain hepatocyte viability and metabolic ability but also faithfully recapitulate the pathophysiological microenvironment of NAFLD observed *in vivo*.

Previous preclinical and clinical studies have elucidated that resmetirom, an agonist of thyroid hormone receptor (THR)-β, is a safe and effective drug for NAFLD and NASH.^[30, 43, 44, 45, 46, 47]^ Resmetirom is an appealing agent for treating NASH because of its impact on lipophagy, mitophagy, mitogenesis, and β-oxidation in hepatocytes. The effects of MSC-Exo on NAFLD were previously reported for their potential to mitigate lipid accumulation.^[48,49]^ However, the therapeutic effect comparison between MSC-Exo and resmetirom has not yet been investigated. The TMSC utilized in this study was the hTERT-driven immortalized MSC established in our lab.^[28]^ This cell line could be used as a highly expandable supply for mass production of MSCs and their exosomes as therapeutic agents. In our earlier study, we found that TMSC-Exo treatment exerts protective effects in acute liver failure.^[28]^ In this study, we investigated the effects of TMSC-Exo compared to resmetirom on NAFLD using the LC model. Our results demonstrated that both treatments effectively alleviated cellular damage and reduced lipid droplet accumulation in hepatocytes. However, compared to resmetirom, TMSC-Exo (at both concentrations of 400 μg/mL and 800 μg/mL) showed superior efficacy in reducing lipid accumulation and improving hepatocyte function. Notably, no significant difference was observed between the high and low doses of TMSC-Exo in improving NAFLD.

To confirm the *in vitro* results, we evaluated the therapeutic efficacy of TMSC-Exo in a DIO-induced NAFLD mouse model, using resmetirom treatment as a positive control. The *in vivo* results showed that both resmetirom and TMSC-Exo improved lipid metabolism and ameliorated hepatic steatosis and hepatocyte damage in NAFLD mice. H&E and Oil Red O staining revealed that the attenuation of hepatic steatosis and lipid metabolism was more pronounced in the TMSC-Exo group than in the resmetirom group. However, there was no significant difference in the quantified positive area of Oil Red O staining between the TMSC-Exo-treated and resmetirom-treated groups, which might be attributed to the limited number of animals per group and individual variability. Additionally, compared to resmetirom, TMSC-Exo was more effective in improving ALT, AST, and TG levels but did not significantly reduce TC levels. Both *in vitro* and *in vivo* results suggest that TMSC-Exo has a strong potential for treating NAFLD. TMSC-Exo demonstrated greater advantages over resmetirom in attenuating lipid accumulation and improving hepatocyte function in the *in vitro* on-chip model. This discrepancy may be due to differences between species-derived models.

The molecular mechanisms underlying the therapeutic effects of TMSC-Exo on NAFLD remain unclear. To investigate the mechanisms of NAFLD development and the effects of TMSC-Exo on NAFLD, proteomic analysis was conducted. Compared to the negative control group, the differentially expressed proteins in the NAFLD group were associated with lipid metabolism (TM199, PLIN2, MRP2)^[50, 51, 52, 53, 54, 55, 56]^ and ER stress (PLP2, SDF2, TMX2).^[38, 57, 58, 59, 60]^ TM199 (also known as TMEM-199) was the most significantly upregulated protein in the NAFLD group compared to the negative control, and its expression was downregulated after TMSC-Exo treatment. TM199 plays a role in maintaining Golgi homeostasis, and its deficiency has been reported to be associated with congenital disorders of glycosylation, hypercholesterolemia, and hepatic steatosis.^[50]^ We hypothesize that the upregulation of TM199 in the NAFLD group and its subsequent downregulation after treatment may represent a cellular protective response. Previous studies have demonstrated that PLIN2 promotes lipid accumulation and contributes to the progression of NAFLD.^[52, 53, 54]^ The expression of PLP2 was also upregulated in mice with NASH.^[38]^ Although PLP2 has been associated with ER stress-induced neuronal apoptosis,^[61]^ its role in NAFLD progression remains unclear. The upregulation of both PLIN2 and PLP2 in the NAFLD group and their subsequent downregulation after treatment suggest that they may be involved in NAFLD development and the therapeutic effects of TMSC-Exo. Other differentially expressed proteins between negative control and NAFLD groups were involved in inflammation (PGH2, A1AG1),^[62,63]^ cytoskeletal reorganization (TPM2, ITB3, DEST)[64, 65, 66], mitochondrial dysfunction (DNM1L, ATP68, COXM1, RT-33, MPC1),^[67, 68, 69, 70, 71]^ hydrolysis of polysaccharides, glycolipids, glycoproteins (NAGA)^[72]^ and endothelin-1(ECE1).^[73]^ TMSC-Exo partially reversed the abnormal expression of several aforementioned proteins (including SDF2, ITB3, DNM1L, A1AG1, MPC1, and MRP2), suggesting their potential involvement in the therapeutic mechanisms of TMSC-Exo. Collectively, our findings suggest that the therapeutic effect of TMSC-Exo on NAFLD is mediated through multiple pathways, including the regulation of lipid metabolism, alleviation of ER stress, inflammation suppression, and modulation of mitochondrial function, which is similar to the mechanisms previously reported for MSC-Exo.^[24, 25, 26]^

This study has several limitations. First, the LC NAFLD model was not organoid-based, and the data may be less reliable than those from an organoid-on-a-chip model for drug evaluation. Second, our culture system lacked the key immune components, particularly Kupffer cells, which are central to the inflammatory response in NAFLD. Future studies should incorporate these cells to better mimic the microenvironment in advanced disease stages. Finally, the molecular mechanisms of TMSC-Exo were not investigated in depth, and the therapeutic effect of TMSC-Exo on NAFLD still requires clinical verification.

## Conclusion

In this study, we established a biomimetic human LC model of NAFLD to comparatively evaluate the therapeutic efficacy of TMSC-Exo *versus* resmetirom. The chip-based NAFLD model successfully recapitulated key disease hallmarks, including hepatic steatosis and functional impairment. Our LC platform is also amenable to generalization for modeling other liver diseases. Notably, TMSC-Exo demonstrated superior therapeutic effects compared to resmetirom in the liver-on-chip system, which was validated in an *in vivo* murine model. These results collectively indicate that: (1) the developed NAFLD-on-chip platform represents a physiologically relevant disease model, and (2) TMSC-Exo exhibits significant clinical potential for NAFLD treatment.
